# Supplementary material for: Intensity scaling of conventional brain magnetic resonance images avoiding cerebral reference regions: A systematic review
Source: PLoS One. 2024 Mar 14;19(3):e0298642. doi: 10.1371/journal.pone.0298642 (PMC10939249; doi:10.1371/journal.pone.0298642)
Supplement: S1 File — Further information on the systematic review process and the included studies. (DOCX) [file pone.0298642.s002.docx]

Supplementary Material

1. **Search strategy and selection process**
   1. **Selection process**

The software Covidence (Covidence systematic review software, Veritas Health Innovation, Melbourne, Australia, n.d.), available online at www.covidence.org, was used for the entire screening and selection process. All identified studies were imported to Covidence. During the import process, the software automatically detected and removed most duplicates (e.g., if multiple databases identified the same study). First, TW and MM independently conducted the screening of titles and abstracts. Disagreements were resolved through discussion. In the next step, selected studies were subject to full-text screening by TW; problematic cases were discussed with MM. No restrictions were applied to publication date and location, and only studies published in English were selected. The reasons for exclusion in full-text screening and the number of studies per reason are presented in *Table 1*. The study of Ali et al. (Ali et al., 2022) reported correlations between different image intensities to be larger or smaller than 0.3 without providing a significance level of the correlations. Additionally, they reported correlations between the different image intensities for 3 subgroups (n=10) individually and for all 30 subjects overall and reported inconsistent results: some correlations (e.g., between T1-weighted (T1w)/T2-weighted (T2w)-ratio and fractional anisotropy (FA)) were reported to be larger than 0.3 in all 3 subgroups by the overall representation (indicated by a solid line in figure 9 (A) of the publication), but the individual presentation of correlations in the subgroups shows otherwise (indicated by solid line for only 2, and not 3, of the 3 subgroups in figure 9 (B), (C), and (D) of the publication). Considering these points, we excluded the study of Ali et al. The study of Kanazawa et al. (Kanazawa et al., 2022) was also excluded because the correlation value reported in the results paragraph was not equal to the ones reported in figure 6 and the discussion of the publication. Hence, it was not possible to reliably extract the data.

*Table 1 Reasons for exclusion in full-text screening*

| **Reason for exclusion** | **Explanation** | **Number of studies** |
| --- | --- | --- |
| other scaling method | The scaling method applied in these studies used cerebral reference regions. | 154 |
| no brain images | The images used in these studies are not brain MRIs (e.g., MRI studies on neck tumours). | 15 |
| no intensity scaling | These studies did not perform intensity scaling. | 14 |
| absence of validation | This exclusion criterion was applied to studies that used T1w/T2w-ratios assuming that it represents myelin or brain integrity without further validation of the method. | 14 |
| no detail about scaling | These studies mentioned the application of intensity scaling or normalization, but did not give methodological details (n=6) or did not assess or validate the scaling method (n=5). | 11 |
| no conventional MRI sequence | Conventional MRI sequences were not used in these studies (e.g., diffusion tensor imaging). | 8 |
| other document type | These two cases were one book chapter and one non-accessible conference paper. | 2 |
| inconsistent results | Results could not be reliably extracted from these studies as reporting was unclear or contradictory | 2 |
| phantom study | An MRI phantom was studied. | 1 |

- 1. **Data collection process**

We extracted relevant data from all studies according to a standardized form. To be included, studies did not have to provide all outcome categories.

1. **Reporting and synthesis methods**
   1. **Outcome categorization**

To report the data, we categorized the studies into the following outcome groups:

• Variance reduction assessment: Studies were attributed to this group if they provided results or qualitative analysis of variance reduction after intensity scaling.

• Comparison to other scaling methods: Studies were attributed to this group if they compared results based on the scaling method of interest to those based on unscaled images or other scaling methods.

• Relation to quantitative imaging: Studies were attributed to this group if they explicitly related or compared results based on the intensity-scaled images to those based on quantitative imaging techniques.

• Relation to clinical or demographical data: Studies were attributed to this group if they related clinical or demographical data and/or subject group differences to scaled image intensities. Only studies having performed analogous analyses with another scaling method, quantitative imaging techniques, or histology for validation were included in this category.

• Relation to histological data: Studies were attributed to this group if they related or compared results based on the intensity-scaled images to those based on histology.

- 1. **Tabulation of results**

As the results of “Variance reduction assessment” and “Comparison to other methods” were commonly jointly reported, we combined these categories. The variance reduction was assessed with different methods and metrics. Hence, methods could only be attributed to three categories of variance reduction (Yes/No/not available or applicable (NA)) or to three categories regarding better performance compared to other methods (Yes/No/NA).

Regarding the “Relation to quantitative sequences” outcome group, we calculated, if applicable, the R^2^ value by squaring the Pearson’s r value in order to make results more comparable to reported R^2^ values of linear (mixed) models.

1. **Risk of Bias in studies**
   1. **Risk of Bias items**

A customized Risk of Bias (RoB)-tool was set up by TW and MM, based on the RoB-tools of Cochrane, QUADAS, and NIH. The following 6 items were included: sample size statement, exclusions, homogeneous method, reference standard, incomplete outcome data, outcome reporting (see *Table 2*). For each study, the risk of bias items were labeled with “low risk of bias” or with “high risk of bias” if the corresponding question could be answered accordingly; otherwise, the items were labeled with “some concerns”. The risk of bias assessment of the studies was conducted by TW, and critical cases were discussed with MM.

*Table 2 Risk of Bias items*

| **Item (original RoB-tool)** | **Description** |
| --- | --- |
| Sample size statement (NIH) | Did the authors report the sample size and whether it was sufficiently large to detect a representative outcome? |
| Exclusions (QUADAS-2) | Did the study avoid inappropriate exclusions and provide reasons for exclusion? |
| Homogeneous method (NIH) | Were other interventions avoided or similar in the groups (e.g., similar background treatments)? (e.g., bias filed correction, preprocessing, scanner type) |
| Reference standard (QUADAS-2) | Is the reference standard likely to correctly classify the target condition? (Is the reference standard reliable) |
| Incomplete outcome data (Cochrane) | Were outcome data available for all, or nearly all, participants? |
| Outcome reporting (NIH, Cochrane) | Were outcomes assessed using valid, appropriate, and reliable measures? |

- 1. Sample size statement

Ten studies were at low risk, while 5 were labeled with “some concerns”, and 9 were at high risk of bias. The 5 studies with some concerns did not explicitly discuss the effect of the sample size on the results, but mentioned that the sample size was larger than in previous studies or “moderately large”, suggesting that studies with larger sample sizes are needed, or had a large sample size (>100) for which a discussion might not be as relevant. The studies with a high risk of bias had relatively small or moderate sample sizes and did not comment on it.

- 1. Exclusion of subjects

Of the included studies, 22 studies were at low risk, and 2 studies were at high risk. The high risk of Brown et al. (Brown et al., 2020) arises because, on the one hand, they provide a detailed description and reasons for exclusion, but on the other hand also excluded “scans exhibiting obvious deviations from participants’ other scans” for intensity modelling without giving the number of excluded scans. Hannoun et al. (Hannoun et al., 2022) only stated that images “with major artifacts” were excluded, without presenting the number of excluded images or specifying “major artifacts”.

- 1. Homogeneous method

Of the included studies, 18 studies were at low risk, 2 studies were labeled with “some concerns”, and 4 studies were at high risk. The study by Zheng et al. (Zheng et al., 2022) used different intensity thresholds for male and female subjects to analyze the images. In another study (Loizou et al., 2009), no information about scan protocols or pre-processing was provided, making it impossible to assess homogeneity and, therefore, we labeled this study with “some concerns”. The four studies at high risk used images from different MRI scanners with different field strengths or images were acquired with different sequence parameters.

- 1. Reference standard

Of the included studies, 22 studies were at low risk, 1 study was labeled with “some concerns”, and 1 study was labeled with “high risk of bias”. Loizou et al. (Loizou et al., 2009) was labeled with “some concerns”, because this study compared many scaling methods to each other, but there was no validated reference standard (such as quantitative imaging). Shim et al. (Shim et al., 2022) compared T1w/T2w ratios to R1xR2* maps (referred to as “q-ratio”: R1 divided by T2* in Shim et al.). In this study, the T1w/T2w ratio, according to the authors, served as reference standard for myelin imaging and was used to validate R1xR2* maps. Hence, we labelled it with “high risk of bias” because they indeed compared T1w/T2w ratios to quantitative imaging (i.e., R1xR2*), but the biological surrogate of R1xR2* has yet to be ascertained, and no validation of the T1w/T2w ratio through comparison with established quantitative imaging techniques was provided.

- 1. Incomplete outcome data

Of the included studies, 19 studies were at low risk, 3 studies were labeled with “some concerns”, and 2 studies were at high risk. Brown et al. (Brown et al., 2020) provided data for all subjects, but used a subgroup of subjects (11-18 years of age) for intensity modelling. This was explained in detail and justified in the study. Luo et al. (Luo et al., 2019) investigated right and left inferior parietal lobe and hippocampus, but correlation results were not reported for inferior parietal lobe when scaled T1w/T2w-ratio was compared to [18F]FDG-PET. The study of Sanada et al. (Sanada et al., 2022) included four different cohorts, but the correlation between scaled T1w/T2w-ratio images and T1- and T2-relaxometry was analyzed in only one of these cohorts (n=8), while the differences between IDH-wildtype and IDH-mutant lower grade glioma was analyzed using the three other cohorts (n=155). Yamamoto et al. (Yamamoto et al., 2022) investigated, similarly to Sanada et al. (Sanada et al., 2022), the relation between scaled T1w/T2w-ratio images and T1- and T2-relaxometry. However, relaxometry data was only available for 2 subjects (total number of subjects in the cohort: 34). In the study of Loizou et al. (Loizou et al., 2009), the Kullback Leibler Divergence (KLD) Distance is reported as a measure of histogram dissimilarity between different slices of the same scan and between slices of different scans. However, this result is only reported for normal volunteers but not for people with MS.

- 1. Outcome reporting

Of the included studies, 19 studies were at low risk, 2 studies were labeled with “some concerns”, and 3 studies were at high risk. Cappelle et al. (Cappelle et al., 2022) was labeled with “some concerns” due to incomplete information. Although it was mentioned in the methods part of the study, no comparison between scan protocols was provided. They compared the difference between scaling methods statistically, but it was impossible to identify which test was applied. In addition, the relation of scaled T1w/T2w and T1w/FLAIR-ratio images with magnetization transfer ratio (MTR) was visually analyzed on a scatter plot without reporting a correlation value, allowing for qualitative but not for quantitative assessment. Yasuno et al. (Yasuno et al., 2017) was labeled with “some concerns” because it was not reported which intensity metric were used for the analysis of group differences (e.g. mean, median). Brown et al. (Brown et al., 2020) presented their correlation analysis results with detailed information about the mixed models; however, in the text, the relationship between intensities and disease duration in normal-appearing white matter (NAWM) and normal-appearing gray matter (NAGM) are reported with different parameter estimates. In NAWM, the interaction term “diagnosis(MS):disease duration” (MS: multiple sclerosis) was used to assess the relationship of intensity and disease duration in MS patients, whereas in the analysis of NAGM the term “disease duration + diagnosis(MS):disease duration” was used. This introduces outcome reporting bias as it is unclear why different terms were used for the same relationship in the mixed models. Furthermore, the variance reduction after scaling was evaluated by visual inspection, allowing for qualitative but not quantitative interpretation. R^2^ values of linear mixed models assessing the relation of scaled image intensities with MTR values were reported without discussing the significance level of the results. After taking these aspects into account, interpretation of results was only partially possible, which led to labeling the study of Brown et al. (Brown et al., 2020) with “high risk of bias”. The correlation between T1w/T2w-ratio values and apparent diffusion coefficient (ADC) values was analyzed by Soun et al. (Soun et al., 2017) through comparison in two very specific brain regions. A linear regression was applied using only the voxel clusters of the two regions, which led to a conclusion about the general relation between T1w/T2w-ratio values and ADC. We labeled this study with “high risk of bias” since correlation analysis of only two clusters, most likely showing highly significant linear correlations, does not necessarily represent the overall correlation between the two measures. The study of Yamamoto et al. (Yamamoto et al., 2022) was also labeled with “high risk of bias”, because 1) probability distributions of high and low Met-PET lesions were said to be different, but this was not supported by statistical analysis, only by referring to the plotted distribution, 2) correlation between T1w/T2w-ratio values and T1- and T2-relaxation values was investigated using only data from two subjects, and 3) a one-sample t-test was used to investigate classification accuracy (area under the curve (AUC) values), but it remained unclear whether AUC values follow the required normal distribution to some degree, which should be assured in a small cohort.

1. **Results of individual studies and syntheses**

The availability of results per outcome group across included studies is shown in *Table 4*. Four studies explicitly investigated variance reduction. Twenty studies related the scaled image intensities to quantitative imaging. Investigation of the relationship between intensity of scaled images and clinical/demographic data was performed by 8 studies. The relation between scaled image intensities and histology was reported in 4 studies.

*Table 3 MRI acquisition settings and bias field correction information*

| Study | Scanner | Field Strength | Sequences | Bias Filed Correction |
| --- | --- | --- | --- | --- |
| Arshad et al. 2017 | MAGNETOM Verio, Siemens | 3T | T1w, T2w, MWF, geomT2IEW | N3 (Sled et al., 1998) |
| Brown et al. 2020 | Twinspeed Excite 12.0, GE Medical Systems | 1.5T | T1w, MTR | N3, MINC toolkit (http://www.bic.mni.mcgill.ca/ServicesSoftware/MINC) |
|  | TIM Trio, Siemens Healthcare | 3T | T1w | N3, MINC toolkit (http://www.bic.mni.mcgill.ca/ServicesSoftware/MINC) |
| Cappelle et al. 2022 | Intera or Ingenia or Achieva, Philips | 3T | T1w, T2w, FLAIR, MTR | ANTs (http://stnava.github.io/ANTs/) |
| Ganzetti et al. 2014 | Gyroscan Intera, Philips Healthcare | 1.5T | T1w, T2w, FLAIR | SPM8 (Ashburner and Friston, 2005) |
|  | Intera, Philips Healthcare | 3T | T1w, T2w, FLAIR | SPM8 (Ashburner and Friston, 2005) |
|  | Achieva, Philips Healthcare | 3T | T1w, T2w, FLAIR, MTR, FA | SPM8 (Ashburner and Friston, 2005) |
| Gilmore et al. 2007 | NA | 3T | T1w, T2w, DWI (FA, MD) | NA |
| Hagiwara et al. 2018 | MAGNETOM Prisma, Siemens Healthcare | 3T | SyMRI Data (T1w, T2w, MVF, MTsat) | SyMRI (Hagiwara et al., 2017) |
| Hannoun et al. 2022 | Sonota, Siemens | 1.5T | T1w, T2w, DWI | ANTs (http://stnava.github.io/ANTs/) |
| Loizou et al. 2009 | NA | NA | T2w | NA |
| Luo et al. 2019 | NA | NA | T1w, T2w, FLAIR, PET imaging | SPM12 (Ashburner and Friston, 2005) |
| Nakamura et al. 2017 | Trio, Siemens | 3T | T1w, T2w, MTR | “in-house” method |
| Pareto et al. 2020 | Tim Trio, Siemens | 3T | T1w, T2w, MTR | SPM12 (Ashburner and Friston, 2005) |
| Preziosa et al. 2021 | Signa-MR750, General Electric Medical Systems | 3T | T1w, T2w, FLAIR | SPM12 (Ashburner and Friston, 2005) |
| Righart et al. 2017 | Achieva, Philips | 3T | T1w, T2w, FLAIR | NA |
| Saccenti et al. 2020 | MAGNETOM Prisma, Siemens Healthcare | 3T | SyMRI Data (T1w, T2w, MVF, MTsat), DWI | SyMRI (Warntjes et al., 2016) |
| Sanada et al. 2022 | 20+ different scanners | 1.5T and 3T | T1w, T2w, T1-relaxometry, T2-relaxometry | SPM12 (Ashburner and Friston, 2005) |
| Shams et al. 2019 | Magnetom Prisma, Siemens | 3T | T1w, T2w, T1-relaxometry | minimal human connectome project preprocessing pipeline (Glasser et al., 2013) |
| Shim et al. 2022 | Magnetom,  Siemens | 7T | T1w, T2*w, T1-relaxometry, T2-relaxometry | NA |
| Soun et al. 2017 | Signa HDxt, GE Medical Systems | 1.5T | T1w, T2w, FLAIR, DWI | Oxford Centre for Functional MRI of the Brain Software Library (http://fsl.fmrib.ox.ac.uk) |
| Uddin et al. 2018 | Achieva, Philips | 3T | T1w, T2w, GRASE (MWF) | SPM12 (Ashburner and Friston, 2005) |
| Uddin et al. 2019 | Achieva, Philips | 3T | T1w, GRASE (T2w, MWF), DWI | SPM12 (Ashburner and Friston, 2005) |
| Vandewouw et al. 2019 | Trio, Siemens | 3T | T1w, T2w, MTR | NA |
| Yamamoto et al. 2022 | Prisma, Siemens Healthcare | 3T | T1w, T2w, T1-relaxometry, T2-relaxometry, [11C]Met-PET | SPM12 (Ashburner and Friston, 2005) |
| Yasuno et al. 2017 | Signa Excite HD V12M4, GE Healthcare | 3T | T1w, T2w, PET imaging | SPM12 (Ashburner and Friston, 2005) |
| Zheng et al. 2022 | Trio, Siemens | 3T | T1w, T2w, MTR | NA |

*Table 4 Data availability of included studies*


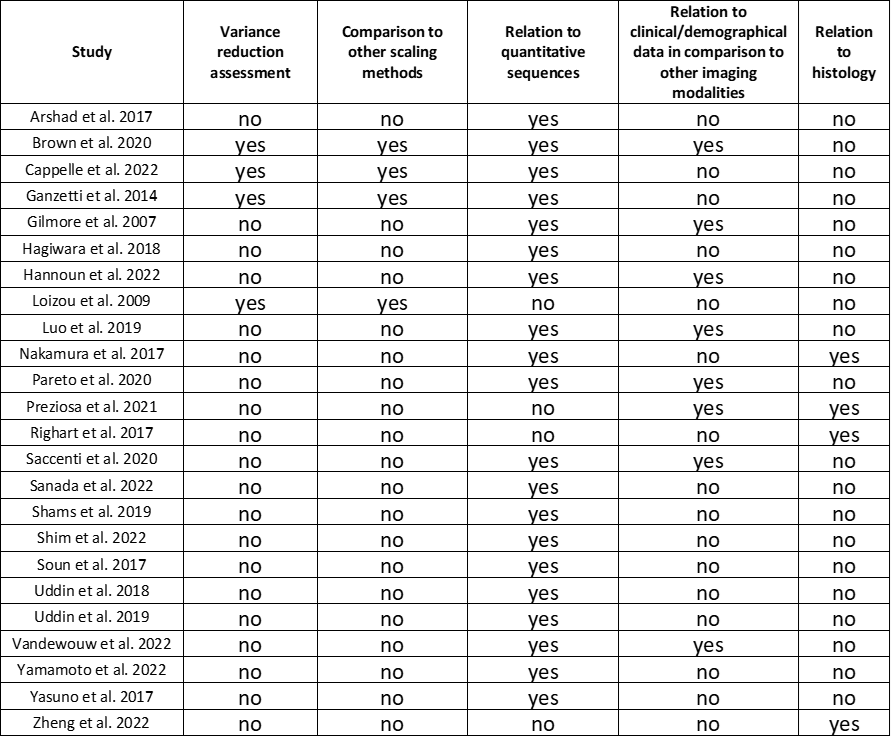


- 1. **Variance reduction assessment and comparison to other methods**

Brown et al. (Brown et al., 2020) found that histograms before scaling exhibited large variability with 4 peaks (i.e., 2 peaks for each WM and GM). After scaling images with orbital fat as reference region, the histogram showed low variability and only two peaks remained (i.e., one peak for each WM and GM). The same was true for normalization with normal-appearing WM (White-Stripe method, introduced by Shinohara et al.). Additionally, scaling images with orbital fat as reference region reduced the inter-scan variance from ~4.5 to ~2 in WM and from ~3.5 to ~2 in GM. Using the White-Stripe method reduced the inter-scan variance even more, namely from ~4.5 to ~1 in WM and from ~3.5 to ~0.5 in GM. We estimated the intensity ranges (given in standardized intensity units) from the intensity histograms, because no value was provided. The values should therefore be considered with an error of +/-0.3 due to possible inaccuracies in the extraction process.

Ganzetti et al. (Ganzetti et al., 2014) reported a decrease in T1w/T2w-ratio variance after scaling the T1w and T2w images. Upon visual comparison of the intensity histogram of T1w/T2w-ratio images using unscaled and scaled T1w and T2w images, the authors concluded that only the histograms of scaled T1w/T2w-ratio images showed similar shapes, intensity scales and inter-subject variability across different datasets (IXI 1.5T dataset, https://brain-development.org/ixi-dataset/; IXI 3T datsaset https://brain-development.org/ixi-dataset/; KIRBY21 3T dataset, https://www.kennedykrieger.org/national-resource-for-quantitative-functional-mri/databases). This finding was confirmed in the WM, in which the T1w/T2w-ratios differed significantly when based on unscaled images: meanIXI1.5T(SD)=3.44(+/-0.31), meanIXI3T(SD)=4.52(+/-0.82), meanKIRBY21(SD)=28.49(+/-4.63), p<0.001; but were similar when based on scaled images: meanIXI1.5T(SD)=2.11(+/-0.15), meanIXI3T(SD)=2.04(+/-0.10), meanKIRBY21(SD)=2.07(+/-0.14), p=0.2236.

Cappelle et al. (Cappelle et al., 2022) is, to the best of our knowledge, the first study that analyzed T1w/FlAIR-ratio images (in addition to T1w/T2w-ratio images). In this study, the scaling method of Ganzetti et al. (Ganzetti et al., 2014) was applied to calculate scaled T1w/T2w-ratio and T1w/FLAIR-ratio images and compared to two other scaling methods: 1) nonlinear histogram calibration using the histogram from extra-cerebral tissue of the ICBM152 template image (https://www.bic.mni.mcgill.ca/ServicesAtlases/ICBM152NLin2009), named NLG calibration, and 2) nonlinear histogram calibration using the histogram from extra-cerebral tissue of a subject template generated from their own population, named NLS calibration. It should be mentioned that, for Ganzetti-scaling and NLG-scaling, the histogram transformation calculated from the T2w image calibration was also applied to the FLAIR image. For the NLG-scaling, an own histogram transformation was applied to the FLAIR image. The coefficients of variance (CoV) of T1w/T2w and T1w/FLAIR-ratios were calculated in NAWM, NAGM, MS lesions, Corpus Callosum, and Thalamus. These values were compared between the different scaling methods as well as with unscaled T1w/T2w and unscaled T1w/FLAIR-ratios. For both T1w/T2w and T1w/FLAIR the CoVs of unscaled ratios were larger than those of any of the scaled ratios, except for the CoVs of the Ganzetti- and NLG-scaled T1w/T2w-ratios in NAWM, which were slightly larger (13.2% and 12.1%, respectively) than the CoV of the unscaled T1w/T2w-ratio in NAWM (12.0%). Only 1 of 10 CoV values of Ganzetti-scaled ratios (scaled T1w/FLAIR-ratio in NAGM) was significantly lower than CoV values of unscaled ratios, whereas 6/10 CoV values of NLG-scaled ratios and 4/10 CoV values of NLS-scaled ratios were significantly lower than CoV values of unscaled ratios. 5/10 CoV values of NLG-scaled ratios and only 1/10 CoV values of NLS-scaled ratios were significantly lower than CoV values of Ganzetti-scaled ratios. Overall, NLG- and NLS-scaling produced lower CoV values than Ganzetti-scaling (applies to T1w/T2w- and T1w/FLAIR-ratios), with slightly lower CoV values when NLG-scaling was applied.

- 1. **Clinical or demographical data and their relation to intensity-scaled conventional MR images in comparison to other imaging/scaling methods**

**Scaled T1w images**

Brown et al. (Brown et al., 2020) observed a trend in that scaled T1w image intensities decreased with age at onset and with disease duration for both monophasic acquired demyelinating syndrome and MS subjects. Generally, intensity changes were consistent with MTR values and with clinical expectations, whereas results based on White-Stripe normalization showed opposite trends compatible with the notion that the region used for scaling had already been affected by the disorder under examination.

The only other study assessing the relation between scaled T1w image intensities and clinical/demographical data was conducted by Gilmore et al. (Gilmore et al., 2007). In this study, gestational age was correlated with image intensities in the genu (central & cortical), the splenium (central & cortical), and the corticospinal tract (central & cortical) using mixed models for each of those regions. A significant correlation between scaled T1w image intensities and gestational age was only found in the central splenium (R^2^=0.1207, p=0.0352, negative relation). Scaled T2w image intensities, as well as diffusion-weighted imaging (DWI)-based values (mean diffusivity (MD) and FA), showed opposite behavior as significant correlations were not observed in the central splenium but in all other regions of interest (ROIs). FA values were positively correlated, while MD values and scaled T2w image intensities were negatively correlated with gestational age.

**Unscaled T1w/T2w-ratio**

Pareto et al. (Pareto et al., 2020) correlated unscaled T1w/T2w ratios within MS-lesions with EDSS (Expanded Disability Status Scale) scores and with disease duration using partial correlation (age and sex as covariates). Significant negative partial correlations were observed for both EDSS (r=-0.66, p=0.003) and disease duration (r=-0.52, p=0.021). When they compared MTR values within MS-lesions with EDSS and with disease duration, the partial correlation was also significant for EDSS (r=-0.64, p=0.004) but not for disease duration (p>0.05).

The relation of development assessments and gestational age with unscaled T1w/T2w-ratios and with MTR values in 4-year-old children (born very preterm and full-term) was studied by Vandewouw et al. (Vandewouw et al., 2019) using simple linear regression. The development assessments were Full Scale Intelligence Quotients (FSIQ), Core Language (CL), and Visual-Motor Integration (VMI). The relations of gestational age with unscaled T1w/T2w-ratios and with MTR values were investigated for very preterm children, but they were not significant. Correlations between unscaled T1w/T2w-ratios and FSIQ, CL, and VMI were all significant in WM (FSIQ: R^2^=0.14, p=0.00731; CL: R^2^=0.13, p=0.0141; VMI: R^2^=0.25, p=0.00012), similar to MTR, which also showed significant correlations with CL and VMI in WM (FSIQ: R^2^=0.05, p=0.10; CL: R^2^=0.10, p=0.0294; VMI: R^2^=0.26, p=0.000111). However, unscaled T1w/T2w-ratios and MTR correlations with development assessments showed different behaviors in subcortical and cortical GM, being significant for T1w/T2w-ratios in subcortical GM and for MTR values in cortical GM.

**Scaled T1w/T2w-ratio**

A recent study by Hannoun et al. (Hannoun et al., 2022) compared scaled T1w/T2w-ratios between healthy controls (HCs) and people with MS in 24 WM ROIs. Compared to HCs, the MS subjects had, on average across all 24 ROIs, a significant decrease in scaled T1w/T2w-ratios of 19.65% (Wilcoxon rank-sum test: p<0.05). These differences went along with significant differences in FA (6.30% decrease for MS subjects), axial diffusivity (AD) (4.76% increase for MS subjects), and radial diffusivity (RD) (10.27% increase for MS subjects).

Luo et al. (Luo et al., 2019) investigated the correlation of scaled T1w/T2w-ratios within bilateral inferior parietal lobule and bilateral hippocampus with neuropsychological data. Specifically, they found significant relations with scores of general cognitive, visuospatial, memory, language, and executive function tests in bilateral hippocampus and left inferior parietal lobule. These results were similar to those based on [18F]FDG-PET images as they found significant relations with the same neuropsychological measures in the same regions. In addition, they found significant relations in the right inferior parietal lobule with scores of general cognitive, visuospatial, memory, language, and attention tests but not with scores of executive function tests.

Saccenti et al. (Saccenti et al., 2020) studied the relations of myelin volume fractions MVF_T1w/T2w_, MVF_SyMRI_, MVF_MTsat_, and RD values with clinical data by exploring the MVF_T1w/T2w_ values in plaque, periplaque, and NAWM ROIs and their correlation with disability (EDSS scores) and disease duration in people with MS. Correlations with EDSS scores could not be found for either imaging technique (p>0.05 in all three ROIs for all four imaging techniques). Only MVF_SyMRI_ (Spearman’s rho=-0.63, p<0.01) and RD values (Spearman’s rho=0.51, p<0.05) in NAWM showed a significant correlation with disease duration.

- 1. **Relation to histology**

Scaled T1w/T2w-ratios were compared to histology-based myelin density (anti-proteolipid protein staining) and neurite density (Bielschowsky silver staining) in the cortex by Preziosa et al. (Preziosa et al., 2021). While they observed a significant correlation with neurite density through linear mixed models (p<0.01), they found no correlation with myelin density. In the (normal-appearing) cortex, they also assessed group differences of histology-based measures (neurite density and myelin density) and scaled T1w/T2w-ratios between healthy controls (n=10) and MS-patients (n=15) using linear mixed models. Healthy controls exhibited significantly higher scaled T1w/T2w-ratios than MS-patients (p=0.045). Histology-based neurite-density values lead to similar results, with healthy controls having significantly higher values than MS-patients (p=0.041), whereas no significant differences were observed for myelin (p>0.05).

Three studies related unscaled T1w/T2w-ratios to histology, all of them within the cortex. Nakamura et al. (Nakamura et al., 2017) used linear mixed-effect models to assess the difference of unscaled T1w/T2w-ratios in histologically confirmed myelinated and demyelinated areas (anti-proteolipid protein staining). Demyelinated regions exhibited significantly lower T1w/T2w-ratios compared to myelinated regions (demyelinated: mean=0.216; myelinated: mean=0.247, p<0.001). They also assessed differences of MTR values between demyelinated and myelinated regions, but the linear mixed model showed no significant difference (demyelinated: mean=36.1; myelinated: mean=37.2, p=0.08).

The relation between unscaled T1w/T2w-ratios and histology (dendrite density: anti-microtubule-associated protein 2 staining, myelin density: anti-proteolipid protein staining, axonal density: anti-SMI312 staining, cortical thickness: measured manually) within the cortex was explored by Righart et al. (Righart et al., 2017) using generalized estimating equations. A significant relation was only found between unscaled T1w/T2w-ratios and dendrite density (p=0.0008).

In addition to unscaled T1w/T2w-ratios, Zheng et al. (Zheng et al., 2022) also related unscaled T2w image intensities and MTR values to histology. Based on the different intensities, the authors aimed to describe myelination in the cortex and compared the results to histology (anti-proteolipid protein staining) by reporting the median values (across subjects) of sensitivity, specificity, and accuracy of detecting demyelinated voxels. Using unscaled T1w/T2w-ratios showed high sensitivity (median(IQR)=0.75(0.37)) but relatively low specificity (median(IQR)=0.13(0.39)) and accuracy (median(IQR)=0.42(0.35)). Similarly, evaluation of MTR values exhibited high sensitivity (median(IQR)=0.78(0.11)) but at the same time relatively low specificity (median(IQR)=0.29(0.22)) and low accuracy (median(IQR)=0.39(0.48)). In contrast, unscaled T2w image intensities showed lower sensitivity (median(IQR)=0.63(0.32)) but higher specificity (median(IQR)=0.46(0.35)) and accuracy (median(IQR)=0.71(0.33)).

*Table 5 Overview of relation between MRI and histology*

Abbreviation: IQR: interquartile range; MTR: magnetization transfer ratio; NA: not available/applicable; ROI: region(s) of interest.

**References**

Ali, T. S., Lv, J., and Calamante, F. (2022). Gradual changes in microarchitectural properties of cortex and juxtacortical white matter: Observed by anatomical and diffusion MRI. *Magn Reson Med* 88, 2485–2503. doi: 10.1002/MRM.29413.

Ashburner, J., and Friston, K. J. (2005). Unified segmentation. *Neuroimage* 26, 839–851. doi: 10.1016/j.neuroimage.2005.02.018.

Brown, R. A., Fetco, D., Fratila, R., Fadda, G., Jiang, S., Alkhawajah, N. M., et al. (2020). Deep learning segmentation of orbital fat to calibrate conventional MRI for longitudinal studies. *Neuroimage* 208. doi: 10.1016/j.neuroimage.2019.116442.

Cappelle, S., Pareto, D., Sunaert, S., Smets, I., Laenen, A., Dubois, B., et al. (2022). T1w/FLAIR ratio standardization as a myelin marker in MS patients. *Neuroimage Clin* 36, 103248. doi: 10.1016/J.NICL.2022.103248.

Covidence systematic review software, Veritas Health Innovation, Melbourne, Australia (n.d.). Available at: https://www.covidence.org/.

Ganzetti, M., Wenderoth, N., and Mantini, D. (2014). Whole brain myelin mapping using T1- and T2-weighted MR imaging data. *Front Hum Neurosci* 8. doi: 10.3389/fnhum.2014.00671.

Gilmore, J. H., Lin, W., Corouge, I., Vetsa, Y. S. K., Smith, J. K., Kang, C., et al. (2007). Early Postnatal Development of Corpus Callosum and Corticospinal White Matter Assessed with Quantitative Tractography. *American Journal of Neuroradiology* 28. doi: 10.3174/ajnr.A0751.

Glasser, M. F., Sotiropoulos, S. N., Wilson, J. A., Coalson, T. S., Fischl, B., Andersson, J. L., et al. (2013). The minimal preprocessing pipelines for the Human Connectome Project. *Neuroimage* 80, 105–124. doi: 10.1016/j.neuroimage.2013.04.127.

Hagiwara, A., Warntjes, M., Hori, M., Andica, C., Nakazawa, M., Kumamaru, K. K., et al. (2017). SyMRI of the Brain: Rapid Quantification of Relaxation Rates and Proton Density, with Synthetic MRI, Automatic Brain Segmentation, and Myelin Measurement. *Invest Radiol* 52, 647–657. doi: 10.1097/RLI.0000000000000365.

Hannoun, S., Kocevar, G., Codjia, P., Barile, B., Cotton, F., Durand‐Dubief, F., et al. (2022). T1/T2 ratio: A quantitative sensitive marker of brain tissue integrity in multiple sclerosis. *Journal of Neuroimaging* 32, 328–336. doi: 10.1111/jon.12943.

Kanazawa, Y., Harada, M., Taniguchi, Y., Hayashi, H., Abe, T., Otomo, M., et al. (2022). Myelin-weighted imaging derived from quantitative parameter mapping. *Eur J Radiol* 156, 110525. doi: 10.1016/J.EJRAD.2022.110525.

Loizou, C. P., Pantziaris, M., Seimenis, I., and Pattichis, C. S. (2009). Brain MR Image Normalization in Texture Analysis of Multiple Sclerosis. in ([IEEE]).

Luo, X., Li, K., Zeng, Q., Huang, P., Jiaerken, Y., Wang, S., et al. (2019). Application of T1-/T2-Weighted Ratio Mapping to Elucidate Intracortical Demyelination Process in the Alzheimer’s Disease Continuum. *Front Neurosci* 13, 904. doi: 10.3389/FNINS.2019.00904/BIBTEX.

Nakamura, K., Chen, J. T., Ontaneda, D., Fox, R. J., and Trapp, B. D. (2017). T1-/T2-weighted ratio differs in demyelinated cortex in multiple sclerosis. *Ann Neurol* 82, 635–639. doi: 10.1002/ANA.25019.

Pareto, D., Garcia-Vidal, A., Alberich, M., Auger, C., Montalban, X., Tintoré, M., et al. (2020). Ratio of T1-Weighted to T2-Weighted Signal Intensity as a Measure of Tissue Integrity: Comparison with Magnetization Transfer Ratio in Patients with Multiple Sclerosis. *American Journal of Neuroradiology* 41, 461–463. doi: 10.3174/AJNR.A6481.

Preziosa, P., Bouman, P. M., Kiljan, S., Steenwijk, M. D., Meani, A., Pouwels, P. J., et al. (2021). Neurite density explains cortical T1-weighted/T2-weighted ratio in multiple sclerosis. *J Neurol Neurosurg Psychiatry* 92, 790–792. doi: 10.1136/JNNP-2020-324391.

Righart, R., Biberacher, V., Jonkman, L. E., Klaver, R., Schmidt, P., Buck, D., et al. (2017). Cortical pathology in multiple sclerosis detected by the T1/T2-weighted ratio from routine magnetic resonance imaging. *Ann Neurol* 82, 519–529. doi: 10.1002/ana.25020.

Saccenti, L., Hagiwara, A., Andica, C., Yokoyama, K., Fujita, S., Kato, S., et al. (2020). Myelin Measurement Using Quantitative Magnetic Resonance Imaging: A Correlation Study Comparing Various Imaging Techniques in Patients with Multiple Sclerosis. *Cells* 9. doi: 10.3390/cells9020393.

Sanada, T., Yamamoto, S., Sakai, M., Umehara, T., Sato, H., Saito, M., et al. (2022). Correlation of T1- to T2-weighted signal intensity ratio with T1- and T2-relaxation time and IDH mutation status in glioma. *Scientific Reports 2022 12:1* 12, 1–9. doi: 10.1038/s41598-022-23527-9.

Shim, J. M., Cho, S. E., Kang, S. G., and Kang, C. K. (2022). Quantitative myelin-related maps from R1 and T2* ratio images using a single ME-MP2RAGE sequence in 7T MRI. *Front Neuroanat* 16, 74. doi: 10.3389/FNANA.2022.950650/BIBTEX.

Sled, J. G., Zijdenbos, A. P., and Evans, A. C. (1998). A Nonparametric Method for Automatic Correction of Intensity Nonuniformity in MRI Data.

Soun, J. E., Liu, M. Z., Cauley, K. A., and Grinband, J. (2017). Evaluation of neonatal brain myelination using the T1- and T2-weighted MRI ratio. *Journal of Magnetic Resonance Imaging* 46, 690–696. doi: 10.1002/jmri.25570.

Vandewouw, M. M., Young, J. M., Shroff, M. M., Taylor, M. J., and Sled, J. G. (2019). Altered myelin maturation in four year old children born very preterm. *Neuroimage Clin* 21. doi: 10.1016/J.NICL.2018.101635.

Warntjes, M., Engström, M., Tisell, A., and Lundberg, P. (2016). Modeling the presence of myelin and edema in the brain based on multi-parametric quantitative MRI. *Front Neurol* 7. doi: 10.3389/fneur.2016.00016.

Yamamoto, S., Sanada, T., Sakai, M., Arisawa, A., Kagawa, N., Shimosegawa, E., et al. (2022). Prediction and Visualization of Non-Enhancing Tumor in Glioblastoma via T1w/T2w-Ratio Map. *Brain Sci* 12. doi: 10.3390/BRAINSCI12010099/S1.

Yasuno, F., Kazui, H., Morita, N., Kajimoto, K., Ihara, M., Taguchi, A., et al. (2017). Use of T1-weighted/T2-weighted magnetic resonance ratio to elucidate changes due to amyloid β accumulation in cognitively normal subjects. *Neuroimage Clin* 13, 209–214. doi: 10.1016/j.nicl.2016.11.029.

Zheng, Y., Dudman, J., Chen, J. T., Mahajan, K. R., Herman, D., Fox, R. J., et al. (2022). Sensitivity of T1/T2-weighted ratio in detection of cortical demyelination is similar to magnetization transfer ratio using post-mortem MRI. *Mult Scler* 28, 198. doi: 10.1177/13524585211014760.
